# The Pathways Undergraduate Researchers Program: Fostering Career Interests, Sense of Belonging, and Student Confidence in Pursuing Science

**DOI:** 10.15695/jstem/v6i2.07

**Published:** 2023-09-26

**Authors:** David Vannier, Beverly Torok-Storb, Shelley Stromholt, Jeanne Ting Chowning

**Affiliations:** 1 Clinical Research Division, Fred Hutchinson Cancer Center, Seattle, WA; 2 Aspect Research + Evaluation, Seattle, WA; 3 Science Education, Fred Hutchinson Cancer Center, Seattle, WA

**Keywords:** Undergraduate Research Experiences, Summer Internships, Belonging, Mentors, STEM, Careers, Biomedical Research, Social and Emotional Support, Underrepresented Minorities

## Abstract

The Pathways Undergraduate Researchers Program is a paid, nine-week summer internship at the Fred Hutchinson Cancer Center. It targets rising first-, second-, and third-year college students from backgrounds underrepresented in biomedical research. This paper describes how the internship impacted students’ awareness of biomedical careers, scientific identification, and sense of belonging in research. Interns reported an increased awareness of biomedical careers and how to attain them. The experience also challenged interns’ career ideas. Interns described a mix of feelings on sense of belonging. All felt welcomed and confident in their abilities. Nonetheless, some noted they were different from the other researchers. A number were motivated by being in the minority and ready to become leaders in diversifying the workforce. Data gathered during the COVID-19 pandemic shed a different light on the internship’s impact. The interns reported becoming ‘credible resources’ on public health issues for their families and communities. The program supported this by building their confidence to understand and communicate science. This undergraduate program developed out of a longer running high school internship effort and many of the strategies described herein are used in both. These findings have implications for programs for underrepresented students at the high school and college level.

## INTRODUCTION

The Pathways Undergraduate Researchers Program (Pathways Undergraduates) is a paid, nine-week summer internship program at the Fred Hutchinson Cancer Center (“Fred Hutch” or “the Hutch”) specifically designed for rising first-, second-, and third-year college students from backgrounds underrepresented in biomedical research as defined by the National Institutes of Health (National Institutes of Health, 2019). The program provides students with authentic research experiences and supports them to make informed decisions about career pathways. The overall goals are 1) to help interns further investigate career paths through immersion in the cultural and scientific practices of biomedicine (Carlone and Johnson, 2007) and 2) to develop a sense of belonging and identification with science that may lead to new possible futures in biomedicine (e.g., Holland et al., 1998; Ribera et al., 2017). Moreover, Pathways Undergraduates seeks to instill students with self confidence that will carry them to achieve as they progress through their career pursuits.

In this paper, we describe the program and present preliminary evaluation findings from the 2018 and 2019 interns, ninety-two percent of whom are from backgrounds underrepresented in biomedical research. Our findings add to previous literature exploring impacts of internship experiences on undergraduates. Studies of these experiences have primarily focused on outcomes such as retention (Nagda et al., 1998); persistence (Jones et al., 2010; Wigfield and Eccles, 2000); self-efficacy (Hunter et al., 2007; Lent et al., 2011, 2013); enjoyment (McKendall et al., 2021); and disciplinary and research knowledge (Parsons et al., 2021). For example, Weston and Laursen (2015) focused on undergraduates’ opportunities to conduct authentic research, design and participate fully in projects, learn basics of scientific methods and skills, as well as the culture and norms of science– all of which contribute to students’ ability to make informed decisions and persist in STEM disciplines. While research has shown that undergraduate research experiences can have positive effects on student attitudes and understanding, impacts on post-graduate and career outcomes are often still contested (see Carpi et al., 2017 for further discussion).

In the design and evaluation of Pathways Undergraduates, we focused on career awareness, scientific identity, and sense of belonging. A focus on career awareness helps fulfill students’ need for “more information and guidance about their distant future” in order to develop effective plans for their future (Zhang and Barnett, 2015). Students’ perceptions of themselves, including self-confidence and science-oriented identification – seeing oneself and being seen by others as a science-oriented person – are important considerations as they make these plans (Carlone and Johnson, 2007; Stet et al., 2016; Syed et al., 2019). Previous studies have found that scientific identity may be the most important factor influencing students’ moves into a science occupation after graduation (Chemers et al., 2011, Estrada et al., 2018; Stets et al., 2017). Scientific identification is also inextricably linked to one’s sense of belonging in that it depends in part on power structures that determine “how we are othered or made to feel we belong” in a community (Le et al., 2019). Sense of belonging includes how students feel they fit in with others, their sense of connection to others and of being valued, both by their peers and the professionals they interacted with during the program (Ribera et al., 2015; Thompson and Jensen-Ryan, 2018).

In our evaluation of the program, we also found that many students in the Pathways program were motivated to pursue a STEM degree or career because of personal concern for health inequities in their communities. Rationale for undergraduate STEM internships has typically focused on economic arguments for broadening participation in STEM; for the US to remain globally competitive, the STEM workforce must be supplied through recruitment and retention of people from groups that have been historically excluded (Carpi et al., 2016; Kendricks et al., 2019; National Academies of Science, 2011). However, research has shown that many students are motivated to pursue a STEM degree or careers out of concern for social justice, and that this concern is often linked to racial/ethnic and gender identities that have been historically marginalized (Carlone and Johnson, 2007; Garibay, 2015; Newman, 2011). Students who do not see STEM as a way to meet their personal and community goals may not persist in STEM degrees or careers (Garibay, 2018). In 2016, the National Academies addressed this by identifying five factors that are important for success in STEM majors, arguing that all students who are interested in STEM majors should: (a) be able to make informed decisions about the best course of study for them based on interests, motivation, and career aspirations; (b) understand the variety of potential career pathways that come with STEM degrees; (c) have a clear understanding of STEM content and practices; (d) not face unreasonable barriers along their pathways that discourage them or make progress impossible; and (e) be aware of connections between STEM and societal issues and concerns (National Academies of Sciences, Engineering, and Medicine, 2016, p.15). Most research on internships has not focused on opportunities for students to make connections between STEM and personal or societal issues and concerns.

We present emergent findings from two cohorts of interns in an undergraduate research experience that inform how we might best support students from historically marginalized backgrounds to participate and persist in biomedicine. Evaluation data collected from 2018 through 2021 from the Pathways Undergraduates program highlights the importance of supporting students in biomedicine through authentic research experiences and meaningful connections to people in the STEM community. These strong social connections have been shown to positively impact undergraduates’ identity and persistence in science (Estrada, et al. 2021). Additionally, our findings suggest that internships could engage students by emphasizing the pursuit of STEM careers as a way to support their social values, such as addressing health disparities. Though the program was not intentionally designed to focus on interns’ societal concerns, the COVID-19 pandemic provided an important and unexpected opportunity for interns to use what they had learned in the internship back in their communities in ways that have implications for future iterations and for STEM internships generally. For example, interns in subsequent years interact with faculty in the Fred Hutch Office of Community Outreach and Engagement and the Office of Community and Government Relations. Here interns are exposed to specific strategies for engaging communities towards better health outcomes.

## PROGRAM CONTEXT

Fred Hutch is an independent, non-profit cancer care and research center in Seattle, Washington with the mission of eliminating cancer and related diseases as causes of human suffering and death. There are over 300 active research groups at Fred Hutch whose interests range from elucidating the mechanisms behind basic cellular functions to developing clinical therapeutics against cancers and studying the community impacts of disease interventions. The Pathways Undergraduates program is run out of the Clinical Research Division but engages faculty and staff from all research divisions across the center.

Pathways Undergraduates was established in 2018 as an opportunity for rising first-, second-, and third-year college students to gain experience in authentic research in a professional setting. The program is funded by the National Cancer Institute’s Youth Enjoy Science R25 mechanism. Interns receive a stipend for the nine-week program to offset lost work opportunities and a free pass for local transportation. Pathways Undergraduates fills an opportunity gap between two longer running programs at Fred Hutch, the Summer High School Internship Program (SHIP) for rising 12th graders and the Summer Undergraduate Research Program (SURP) for fourth-year college students. While these programs are complementary, they are not designed to be a track for individual students. Pathways Undergraduate Researchers was developed by the SHIP faculty and program staff. Fifty percent and thirty percent of the 2018 and 2019 undergraduate cohorts previously participated in SHIP, respectively. Only one Pathways Undergraduate intern from these cohorts went on to participate in SURP.

Pathways Undergraduates is a nine-week program specifically designed for students from backgrounds underrepresented in biomedical research. Interns come from a variety of two- and four-year colleges and have varying levels of STEM exposure and knowledge. While applicants come from across the country, program staff partner with organizations that serve minority students in western Washington State to recruit participants. These organizations include MESA and TriO programs in Washington State community colleges, Rainier Scholars, El Centro de la Raza, and Washington State Opportunity Scholarship. Rather than focusing on grades and test scores, the internship application and selection process take a holistic approach to identifying students with potential, determination, and a genuine enthusiasm to learn. Short essays on the application ask students to give their reasons for coming to Fred Hutch, detail how they will contribute to the overall intern community and describe challenges they are facing or have faced. Intern finalists are interviewed to determine their scientific and career interests and to get a sense of their personalities. Pathways Undergraduates staff use this information in pairing interns and assigning them to faculty research groups.

The program design is based on three elements—scientific, professional, and social/emotional—that work synergistically to prepare students for success in their academic and career goals. The summer program timeline and elements are described below and are summarized in Table 1. The Pathways Undergraduates program and the Summer High School Internship Program (SHIP) are run by the same staff, and the programs share these three overarching elements. An exception is that the professional development activities in SHIP focus on college entrance and success, while the undergraduates concentrate on careers and their attainment.

### Summer Week 1: Orientation and Training for Successful Entry into Fred Hutch Research Groups.

Students prepare for their research experiences during the initial week of the summer. Interns are introduced to Fred Hutch culture and history, and the professional research environment. They learn basic laboratory skills, responsible research protocols, and safety in dedicated training labs. Interns are introduced to computational biology methods in the Fred Hutch computer lab. They also meet with their faculty mentors to review mutual goals and expectations. Many interns do not have laboratory experience outside the classroom and the first week provides a foundation of skills and knowledge. More importantly, the first week activities allow interns to start bonding with one another and form a cohesive, supporting cohort. During the first week and throughout the summer, returning interns serve as peer-peer mentors in preparing first-time participants for their research experience in a faculty lab or computational biology group.

### Summer Weeks 2–9: Mentored Research and Internship Programming.

The bulk of the interns’ time from the second week onward centers on mentored research with Fred Hutch faculty. Interns are paired and placed into mentor research labs full time, Mondays through Thursdays. Based on the interns’ interviews, program staff pair students with similar interests and match them with an appropriate research group. Peers working together are able to support each other and construct a deeper understanding of their laboratory research experience through discussion. Once their research assignment is made, training requirements specific to that lab are addressed, such as Institutional Animal Care and Use Committee (IACUC) training, if necessary. Mentors and interns work collaboratively to develop a project that is of mutual interest, scientifically meaningful, and appropriate in scope. Interns are typically supervised by a senior graduate student, postdoctoral fellow, or staff scientist. Scientist participants are guided by a mentoring expectations document developed by program staff. Interns also receive a complimentary internship expectations document and are briefed on it during the orientation week. Program staff visit labs intermittently to ensure satisfactory progress and are available throughout the summer to support interns in their research projects.

#### Scientific Programming.

Formal programming sessions are held over lunch two days a week and all day on Friday. These activities complement the research experiences and provide interns with exposure to the full range of people and studies at Fred Hutch. To advance their scientific knowledge, interns participate in numerous sessions on cancer research from internationally renowned faculty. Specific topics vary from year to year, but general areas include the molecular and cellular mechanisms of cancer, clinical diagnosis and treatments, and population dynamics and health disparities. In addition to addressing the science and methods, all speakers share their backgrounds, their inspiration for becoming scientists, and their advice for interns. These personal revelations are often impactful with the interns in building a bond with the speakers. During lunch-time sessions, interns meet for casual conversations with Fred Hutch staff scientists, post-doctoral fellows and University of Washington graduate students. The speakers are typically closer in age to the undergraduate interns and provide a “boots on the ground” perspective of biomedical research. The interns gain knowledge of the science and methods used in various groups but also a perspective of the day-to-day events in conducting research at a cancer center. These sessions also touch on mentorship and making the most of the summer experience beyond the bench.

#### Professional Development.

The undergraduate students are keenly interested in gaining the skills and knowledge needed to secure a job in research, health, and medicine-related fields. Fred Hutch scientists and staff are enlisted to raise interns’ awareness of the range of occupations and qualifications needed to succeed in health and medicine. Specifically, a sequential series of one-hour discussions address topics such as Who’s Who in the Research Lab, Life as a Research Scientist, and The Path to Becoming a Physician-Scientist. During ninety-minute career panels interns hear from professionals in a specific realm of biomedicine. Whenever possible, panelists come from backgrounds underrepresented in biomedical research and describe their career and personal journey. Student questions drive the panel discussions and opportunities for one-on-one meetings are set after each session. Panel topics include Biomedical Research Careers, Careers in Health and Medicine, Careers in Health Disparities and Community-based Research, and Biotechnology Industry Careers.

A separate series of workshops focuses on the nuts and bolts of attaining biomedical careers and entering graduate degree programs. Human Resources (HR) staff describe the Fred Hutch employment process from developing the initial job posting to selection and hiring. An HR recruiter describes how to develop a successful resume and provides opportunities afterwards for interns to get personalized resume advice. Program staff run sessions on developing a personal statement towards attaining a research internship in the following summer. Finally, interns engage in a workshop led by program staff on the elements of successful scientific presentations.

#### Social and Emotional Development.

The third component of the program is building the social and emotional well-being of the interns to support their success in their academic and career pursuits. Ninety-two percent of the interns are from backgrounds underrepresented in biomedicine and we enlisted speakers who reflect on their experiences as URM scientists. Topics included discussions on imposter phenomenon (Clance and Imes, 1978), a Fred Hutch professor’s experience as a Latino MD/PhD oncologist and a young African-American physician’s career pathway in research and health advocacy. These speakers serve as role models for the Pathways Undergraduate interns. In turn, the undergraduate interns serve as near-peer mentors to the high school participants in the concurrent SHIP internship program. Throughout the summer the undergrads meet with their younger peers to discuss college selection and preparation. Finally, the program coordinates several social outings for the entire student cohort to build comradery and community during the summer. Interns are encouraged to take initiative and plan their own social events from meeting over lunch to weekend excursions across Seattle. Participant relationships established over the nine-week internship often extend well beyond the summer, and the intern alumni form bonds that continue for years.

Pathways Undergraduates has a formal arrangement with the Molecular and Cellular Biology (MCB) PhD program at the University of Washington. A majority of the graduate students in laboratories at Fred Hutch are enrolled in the MCB program. These graduate students may choose to complete one of their two teaching assistant requirements with the internship program during the summer. Teaching assistants’ duties include supervising interns during the first week of orientation in the training labs and leading sessions on research techniques and practices, such as the basics of PCR and how to read a scientific paper throughout the summer. Beyond being instructors, the MCB students serve as near-peer mentors. They assist in the career goals of the Pathways Undergraduates program in describing the logistics and firsthand experience of graduate studies. Finally, they also aid in the Social and Emotional Development goals by providing support to interns as they proceed through the summer research program.

The summer program ends with an Internship Symposium where students present their research to the Fred Hutch community. Intern pairs present their research projects, describe how their work fits into the overall interests of their host lab’s focus and reflect on their growth during the summer experience. The interns’ mentors and program staff assist the interns in developing presentations that are detailed yet appropriate for a general audience. The final presentation also provides the interns with a formal opportunity to reflect on their nine-week experiences.

The relationships between undergrad interns and Fred Hutch scientists and staff does not end at the conclusion of the summer. When circumstances permit, undergraduate interns have stayed on in the research labs of their faculty mentor. Program staff are also available to support alumni with advice and recommendations as they advance in their academic and career pursuits. All internship alumni are invited to the Fred Hutch campus for a well-attended reunion at the end of the calendar year. Table 1 outlines the support provided interns during the academic years following the initial summer internship.

##### Program Design.

The design of Pathways Undergraduates centers on career awareness, scientific identity, and sense of belonging. The program elements above align to support these goals in the following manner:

#### Career Awareness.

The interns spend eight weeks immersed in a cancer research group and through daily interactions, learn the career pathways and goals of their host mentors. Other careers are explored through seminars and workshops from people in the wide variety of health and medical careers represented at a cancer research center. Interns are also prepared and encouraged to contact Fred Hutch faculty and staff for information interviews during the summer and afterwards.

#### Science Identity.

Pathways Undergraduates relies heavily on establishing a supportive community of interns, all of whom are deeply interested, if not identifying, in science. Ninety-two percent of the 2018 and 2019 interns were from backgrounds underrepresented in science. During the cohort activities and in interactions around campus, interns literally see themselves in science and reaffirm each other’s presence at an internally recognized biomedical research center. Unfortunately, it is difficult for interns to interact with Fred Hutch researchers who are also from URM backgrounds. The Fred Hutch 2021 Diversity Equity and Inclusion Annual Report found that only 4% of faculty and 6% of postdocs and staff scientists meet the NIH-defined URM criteria (Fred Hutchinson Cancer Center, 2021). To overcome this obstacle, all Fred Hutch personnel who interact with interns are encouraged to share the struggles in their science careers and research. This approach draws from the observation that students are motivated to learn science when they hear how famous scientists struggled (Lin-Siegler et al. 2016).

#### Sense of Belonging.

Instilling a science of belonging is infused across all elements of the program. Community building aspects and the lab experience work to connect interns with each other and the professionals in their research settings. It is difficult to identify which program components specifically instill a sense of belonging for any given interns. This topic will be addressed in future evaluation efforts.

## PARTICIPANTS

In the first two years of the program (2018 and 2019), 49 individuals participated in Pathways Undergraduates. Twenty-six (>50%) returned after their first year and spent two or more years as an intern at Fred Hutch and 92% of the participants were from backgrounds underrepresented in biomedical research as defined by NIH. Further demographics are detailed in Table 2.

## EVALUATION METHODS

Evaluation of the Pathways Undergraduates program has focused on student awareness of biomedical careers and development of science identity and sense of belonging. Before the COVID-19 pandemic, the evaluation drew on focus groups with both new and returning interns (in 2018 and 2019) as well as yearly online surveys. These in-person focus groups were conducted during the final week of the program and reflect the interns’ experiences at that time. In these data sources, interns reflected on the outcomes of the program and provided feedback. In March 2021, in response to the COVID-19 pandemic, the evaluation shifted to learn more about the impacts of the pandemic on the previously identified outcomes of the internship program. To this end, a third focus group was conducted virtually with 2018 and 2019 interns who were then program alumni. The 19- and 31-month gaps between the end of the internship and this focus group allowed us to gain insight into the longer lasting effects of the program. In all three focus groups, interns/alumni self-selected based on interest and availability and were modestly compensated for their time. In December 2021, a follow-up survey was distributed to all alumni that focused on themes that arose in the alumni focus group to gauge how widespread the findings related to the pandemic were on alumni perceptions of their internship experience in regard to communicating health information to their families and communities. A description of the evaluation participants and each data source is in Table 3.

### Analysis.

We analyzed Likert-scale survey data (3-point) using descriptive and inferential statistics using McNemar’s test of paired dichotomous data to determine the distribution of response. Before analysis, we re-coded the data to make it dichotomous by bucketing the lower two categories together in order to focus on the number of students who gave the highest rating for each item. Here we report the frequency of “high” ratings, as well as the significance of the findings for each item.. We analyzed qualitative data from open-ended survey responses and focus group transcripts using a grounded theory approach to identify patterns and generate general themes. Afterwards, we identified representative quotes for each theme (Strauss and Corbin, 1997). Some quotes have been edited for clarity.

### Limitations.

There are several limitations to this study. First, Pathways Undergraduates is an authentic research experience internship designed to raise awareness of research and research careers, the pathways to attaining these careers, and what it takes to succeed. While current programming touches on the topics of health equity and community engagement, these were not been the primary focus of the program in 2018 and 2019. Second, in this preliminary evaluation, the intern survey responses from two cohorts were anonymous in order to encourage interns to share their thoughts freely and gather a wide range of possible responses - this means that we did not track unique participants in each evaluation moment. The focus groups were intended to facilitate a dialogue that allowed participants to engage with each other and build on each other’s insights. Finally, the findings presented did not necessarily apply to each participant but were broadly relevant across our students. The themes surfaced in our evaluation are generalizable with respect to setting, educational intervention, and materials. The feedback received from interns and alumni provide important insights into the potential benefits of undergraduate research internships and direction for future program design and evaluation.

## RESULTS

Pathways Undergraduates participants described how the internship provided them with opportunities to develop their a) awareness of and interest in cancer research related careers, b) sense of belonging as scientists, and c) confidence in their ability to succeed in a biomedical career. Additionally, alumni described the important ways in which the undergraduate research experience supported both their motivation to address health disparities and their sense of belonging in biomedicine during the COVID-19 pandemic by d) helping them to become credible resources in their home communities. Below, we describe these findings in more detail.

### Interns Reported Increased Awareness of and Interest in Biomedical Careers.

**Figure 1.**
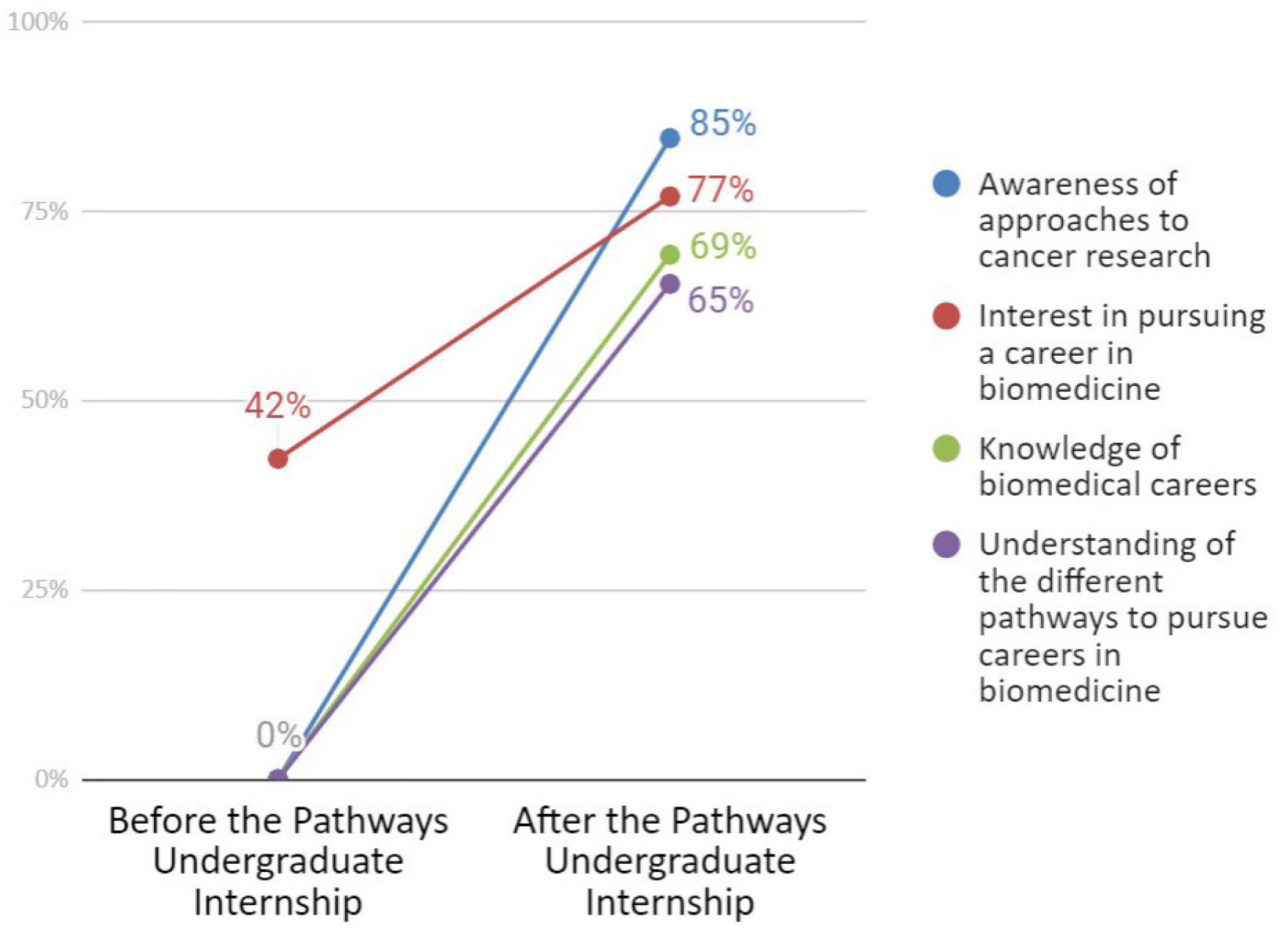
2020 Pathways Survey Results: Career awareness and interest - Percentage of respondents who indicated “high” or 3 on a scale of 1 to 3. Spring 2020, n=26

Results from the March 2020 retrospective pre/post survey (n=26) show that the percentage of interns who indicated “high” ratings (scale 1–3; low to high) increased substantially for each item related to career awareness and interest (Figure 1). Before the internship, no interns indicated a rating of “high” for their awareness of approaches to cancer research, their knowledge of biomedical careers, or their understanding of the different pathways to pursue careers in biomedicine. After the program, 65% of interns or more indicated a “high” rating on each item; for example, 85% indicated a high level of awareness of approaches to cancer research. Perhaps unsurprisingly, interns started the internship with a somewhat high rate of interest in pursuing a career in biomedicine (42%), but they still reported important gains over the course of the program, with 77% reporting a high level of interest after the internship. All four pre/post items exhibited a statistically significant change when analyzed with a McNemar’s test (p < 0.05 for all items). The March 2020 survey also found interns were already interested, or became interested, in medical professions ranging from being a physician’s assistant to a neonatologist.

### The Internship Informed Students’ Academic Choices and Career Navigation.

One of the most immediate results of program participation was how the experience influenced interns in their academic choices while they were still in the internship. In the 2018 and 2019 focus groups, returning interns reported on how they changed, or planned to change, their course selections as a result of their internship experience. Interns added courses in subjects that they had just learned about in the internship, such as epidemiology, medical anthropology, computational science, and environmental health; changed from calculus to statistics; and talked about the value of enrolling in a year-long biology sequence instead of taking electives.

I wanted to be a radiologist…after the program I think I would kind of like to work with viruses. It is so interesting how they invade and mutate.

(First Year Intern, 2018 focus group)

The Fred Hutch internship has been one of the most amazing and changing experiences I have had. Before this experience, my interest in science was high but I never thought I would love being involved in research, and it completely changed my mind. I also discovered a new area of science that I fell in love with, a pathway that I never even knew really existed before, which is the field of public health.

(Alum, 2021 survey)

The opportunity to engage in authentic research projects played a large role in interns’ future career considerations. Interns reported that the program enabled them to try out different components of research work and identify what they did and did not want to do – in both specific tasks or lab work, and more generally in areas of study. The experience motivated some interns to seek out a research career, as described by one second year intern in 2019.

*This* [research experience] *completely solidified my interest in science. I was kind of on the fence about it, I didn’t know if I wanted to work with kids or if I wanted to work in a lab, or if I want to do this or that. Last year I was trying to keep up, but because I’ve gained knowledge through my classes and through my last year, I was able to actually understand why we’re doing what we’re doing, and that got me going with confidence as well, believing in myself, and knowing that is the path. And I would not have known that had I not been here.*

(Returning Intern, 2019 focus group)

In addition to the research itself, interns recognized and appreciated the unique opportunities in the internship to interact with Fred Hutch scientists through seminars, career panels, and casual lunch sessions. In focus groups, many interns described the breadth of career possibilities they learned about at the Hutch that they were not previously familiar with, such as working in global health, public health, and computational biology. In addition to being better informed of the range of career options, interactions with Fred Hutch professionals gave the interns important understandings of what it takes to enter various biomedical careers— such as the time commitment to become a research scientist or an MD/PhD. Some interns reported that this increased awareness also helped to increase their confidence in working towards their goals. One intern described how these connections to professionals made a difference in their experience:

I’m a first-generation college student. So, there’s no one there to tell me what I need to do to get to my final goal…how long I’m going to be in school…. I am Hispanic and I didn’t realize that when you go toward an education for a really long time you have to sacrifice…. We had different speakers and some who really interested me so I emailed them. They were all really happy to get back to me and emailed me back the same day.

(First Year Intern, 2018)

Additionally, some alumni reported that their experience helped them at junctures such as making the transition from community college to a four-year institution, or deciding to pursue research opportunities and college majors that they would not have otherwise. Alumni reflected on a variety of other ways the program supported their academic and career navigation since their internship. They described the impact of the program on their success in college science courses, learning to work in a team and other job skills, and how to think critically. As one participant said, the program helped with “academic challenges in school, finding the gaps in the information I am given, thinking critically about my surroundings, and feeling comfortable in challenging new situations.” Another noted that the program taught both career-related and life skills, in that it “taught me how to read a [scientific] paper and how to take a bus—both essential skills.”

### Interns Felt a Sense of Belonging at Fred Hutch and Increased Identification as Scientists.

Interns generally felt warmly welcomed at Fred Hutch and felt they had opportunities to build a strong sense of community with their peers, lab staff, and mentors. Interns described examples of interactions with mentors, specific aspects of their lab experiences, finding scientists who were open to networking with them, and even those who told them what to see in Seattle. Interns described Hutch staff and mentors using terms such as “patient,” “supportive,” and “encouraging.” Interns described feeling comfortable enough to reach out to mentors and others to discuss personal concerns about their career paths and work/life balance or emailing professionals in other labs to ask about their work. These connections helped many interns feel comfortable in the internships and identify their future place in biomedical research.

I felt comfortable just because there was so much diversity in my lab. My lab mentor has a different ethnicity than I, but we actually bonded with her because we had so many similarities in our culture… We all come from different backgrounds, and so I think that I was fortunate enough to have a lab that was very diverse and a really good mentor. That helped me feel welcomed.

(First Year Intern, 2019 focus group)

Interns benefited from hearing that their mentors, lab team members, and fellow interns had similar backgrounds and experiences to them–the interns who experienced this felt seen and understood because of those similar experiences and values. One intern described how supported they felt with a mentor close to them in age, who was able to empathize with the intern and share their similar experiences as a younger researcher. Interns appreciated the opportunities they had to better understand who scientists are and their stories, which helped the participants to see scientists as approachable and increase their sense of connection to the program. Another intern described the impact of hearing a Program Director describe coming from poverty and experiencing imposter phenomenon (Clance and Imes, 1978). These kinds of experiences with more senior researchers helped interns see themselves in the same earlier career stages their mentors had been through and they were then able to see their future selves in their mentor’s position. Additionally, being part of a cohort of interns from similar backgrounds was also important to many interns. In 2018 and 2019, interns reported that they appreciated the time their cohort of peers was able to spend together where they felt they could be vulnerable and ask “dumb” questions: “Just having those gatherings where all the interns can get together.…Doing the icebreakers and really getting to know each other. Makes me feel like there are other people like me, maybe I do belong here” (First Year Intern, 2018 focus group).

All of these connections served to reinforce many interns’ identities as scientists and part of the Fred Hutch community.

I thought this internship was really valuable in determining “Do I want to go into STEM or not?” Because prior to this internship I was not sure if … someone from my background would be able to do something like this. And so, I feel much more confident in my STEM classes I am enrolled in for the fall. I am looking forward to reaching for that goal.

(First Year Intern, 2019 focus group)

Not all interns had this same experience, especially those who identified as the only person of color in their lab. Some interns described finding it difficult to connect with mentors who were frequently absent from the lab or did not provide feedback, as well as the anxiety some interns experienced as the only URM in the lab. In the focus groups, this did not seem to negatively impact interns’ sense of belonging but strengthened their interest in moving forward in the field to serve as representatives for younger people who will follow them. This aspect of the program may warrant further examination.

*I think* [being the only person of color] *just motivates me. They’re (people of color) not here now, but that doesn’t mean they won’t be here any other time. Maybe you could be that person that adds it, you know?*

(Returning Intern, 2019 focus group)

### Interns Reported Increased Confidence in Their Ability to Succeed in a Biomedical Career.

Intern confidence emerged as an important factor in intern sense of belonging at the Hutch, their identification as scientists, as well as their ability to navigate academic and career decisions. For example, “My experience at the Hutch was very helpful in giving me the confidence to put myself out there in different science areas at my college and applying for different things, just in general” (Alum, 2021 focus group).

Interns attributed some of this increase in confidence and a resulting increased sense of belonging to the reciprocal trusting relationships built in the internship. Interns felt trusted to carry out their work autonomously in the lab, given real responsibilities, and had the opportunity to make important contributions. At the same time, they were able to trust their mentors to be available and supportive. In some cases, trust was facilitated because of the shared cultural experiences interns had with their mentors and others at the Hutch, even if they were from different cultural backgrounds: “The internship program showed me that I belong in biomedical research and that I have the capability to succeed” (Alum, 2021 survey).

In one example, a returning intern noted an increased confidence about reaching out to meet with researchers after initially being afraid to ask. The experience of initiating meetings with scientists to learn about their research had an important influence on the intern’s career plans. Another intern described how the research experience provided opportunities to learn and develop new science skills that increased their confidence and affected their identity as a scientist:

So, for me, I had no research experience prior to this and so doing this just made me be more confident in my science skills because I was able to use what I learned in my general chemistry classes and apply that math to my work and also applying the biology that I know and it really helped me … grow as a scientist. And I am like ‘Wow, I really am a scientist now.’ I did not feel that while doing my science classes but doing it here and being able to apply my knowledge, I really do feel very smart.

(First Year Intern, 2019)

In the later alumni focus group, the impact of the Pathways Undergraduates experience on their confidence in research skills was still evident; alumni talked extensively on how their confidence in their skills gained during the internship continued to impact their professional paths– including their ability to communicate contemporary science, carry out benchwork, ask for help, and speak publicly. Some described how after a few weeks in the internship they were given full responsibility and freedom to do the protocols they were given. For them, this trust had a direct impact on their confidence in college lab classes, and their identification as competent contributors to science, capable of taking the next steps in their career pathways.

…[A]*t first, coming in I was like, man can I…be a scientist? Can I do anything? Am I smart enough? For them to…give me that full freedom and just do my thing, it shows that I can follow that protocol through. That gave me a lot of confidence…* [Later, in my last year of college] *I didn’t feel the need to ask the lab tutor a bunch of questions. …I just kind of look over everything and go ahead and do my experiment. So I felt like I can be somebody who can contribute to science…*

(Alum, 2021 focus group)

### Reflections on the Internship in Light of the COVID-19 Pandemic.

In early 2021, program alumni reflected on experiences that shaped their sense of belonging to the biomedical community, including their personal experiences with the COVID-19 pandemic, how the field has addressed the pandemic, the disparate outcomes for marginalized communities, and their relevant experience with the Pathways Undergraduates program. Health disparities motivated many interns to pursue biomedicine; the pandemic strengthened this motivation. Alumni described how even before the pandemic, structural inequities in the medical field strongly influenced their interest in and sense of belonging to the biomedical community. In their original applications to the program, over a third of the interns described personal experiences with health disparities as a motivating factor to pursue a career in health and medicine. Alumni described this motivation in more detail in the focus group. One said that their decision to study health policy was based on “adverse experiences with the medical establishment, and my family’s own adverse experiences with it, especially because my grandmother recently passed.” Another said, “seeing that social justice could be applied to the medical field in that there’s health disparities everywhere that aren’t addressed on a regular basis, was something that also attracted me to the medical field.” The pandemic made their potential role in addressing these disparities central for all of the participants in the focus group.

### Alumni Reported that the Internship Helped Them Become Trusted Sources of Health Information.

Alumni reported that in part because of their involvement in the program, they took on important roles as credible resources on public health issues for their families and communities during the COVID-19 pandemic, which positively impacted their sense of belonging to the field. The confidence-building experiences of the Pathways Undergraduates program described earlier in this paper contributed to their positioning as science communicators and experts, by themselves and by their families/communities. Specifically, the program supported their confidence to find, understand, and explain complex, personally-consequential public health research—this case scientific research on the COVID-19 vaccine and other public health measures.

At the Hutch when I was able to see how the clinical trials were conducted, and understand the processes and learn more about that and HIPAA, it made the development of the vaccine, although it was later in the stage of the pandemic, I understood that more and was able to communicate those steps more, to my community and family members who normally wouldn’t be as likely to get the vaccine, so I felt like that was really beneficial for me.

(Alum, 2021 focus group)

Almost all the alumni in the focus group took the opportunity to share stories in which they identified as members of multiple overlapping communities—their home communities, which have often not been sufficiently served by the medical community—and as science-oriented individuals in the science community. Alumni described how the disparate health outcomes of the pandemic have been ongoing and have impacted them personally. Because of their unique positioning in different communities, they described themselves as having a crucial role as credible sources, and potentially as advocates, in addressing inequities. One alum said:

My family is still not convinced to take the vaccine yet, and I have to communicate with them and as a first year student…I felt like I was responsible to educate them. ‘Cuz they’ve been fed a lot of information that is conspiracy theories, about having a chip in your arms, and stuff, and they kind of believe that.”

(Alum, 2021 focus group)

This student added that they helped their family members identify valid sources of health information. Another commented:

My parents are…eligible for getting the vaccine so they have many questions that they ask me like “Hey is this like legitimate and is it safe? What are other things that we need to be concerned about?”, so I think because of the skill that I obtained from the Fred Hutch and also from my classes I was able to look through scholarly articles and the trials online like W.H.O. or other sources and I was able to explain to them and I said, “It’s safe, you should get it because it’s good for your community so we can get it. We can reduce this pandemic as soon as possible because we are tired of this.”

(Alum, 2021 focus group)

A follow-up survey was sent to the broader alumni group to try to determine how widespread these experiences were during the pandemic. All respondents (n=6) indicated that their Hutch experience influenced their ability to make sense of the vaccine development process and the pandemic generally, in ways that made them more confident in their ideas, choices, and advice to others. Some respondents reported that the pandemic made them more aware of actions they could take to keep their community safe, such as preparing for resurgences and helping to make sense of conflicting or varying public health policies.

## DISCUSSION

The Pathways Undergraduates program provided important opportunities for interns to learn about and gain further interest in a wide variety of biomedical careers, which in turn shaped their academic choices and career navigation as they plan for their futures. Many felt supported by staff and the research environment, developing their identification as scientists, a sense of belonging in biomedicine, and their confidence in their ability to succeed. Additionally, several alumni described how their experience in Pathways Undergraduates contributed to their ability to find and make sense of, and communicate about complex, personally consequential public health research with and for their home communities. The program made them more knowledgeable about pursuing certain career pathways and supported specific skills that they have used to address health inequities and help others in response to the onset of the COVID-19 pandemic.

Similar to other undergraduate research experience (URE) programs (reviewed by Ahmad and Al-Thami, 2022 and National Academies of Sciences, Engineering, and Medicine, 2017), Pathways Undergraduates provides students with an intensive, authentic experience under the mentorship of a Fred Hutch faculty member. The research experience is supplemented by programming aimed to build students’ scientific, professional, and social/emotional abilities to succeed. Importantly, there are several unique aspects to Pathways Undergraduates compared with traditional UREs that may impact the interns’ experiences. First, the Fred Hutch is not an undergraduate institution. Second, in addition to coming from underrepresented backgrounds, the interns are often the youngest members of their research group. Further, we target first- and second-year college students, while most internship programs select upper-level undergraduates with research experience and advanced coursework (Ahmad and Al-Thami, 2022).

To address these potential points of isolation, interns are supported by a multi-tiered mentoring system (Huerta et al., 2022). Interns are placed into labs in pairs where they serve as peer-peer mentors to each other. The entire internship cohort meets frequently and engages in activities specifically designed to build a strong support network. Graduate students from the University of Washington serve as teaching assistants and near-peer mentors throughout the summer. Finally, the undergraduate interns act as role models and near-peer mentor to the high school students in our concurrent summer program. Estrada et al. (2021) stress the importance of building strong social relationships in supporting undergraduates to succeed in STEM. Pathways Undergraduates seeks to establish social connections and raise awareness among interns of their importance in career persistence.

Though it was not originally designed with this intent, the Pathways Undergraduates internship provided opportunities for interns to “move beyond academic achievement” and “cultivate their commitment to their communities and cultures of origin” (Morales-Doyle, 2017). Many interns were originally motivated by community issues, such as health inequities, to pursue specific career paths. In addition to biomedical research knowledge and experience, the internship served to reinforce how they, and others, position them as credible resources (Morales-Doyle, 2017), in that interns described being health advocates by helping their families make evidence-based decisions to protect themselves and their communities at the start of the COVID-19 pandemic.

In response to these observations, subsequent years of the Pathways Undergraduates program have focused on improving support for interns to become more aware of connections between STEM and societal issues and concerns (National Academies of Sciences, Engineering, and Medicine, 2016), including an emphasis on the health disparities work at Fred Hutch. These developments are motivated by a desire to make training and retention of students going into biomedical careers more equitable by encouraging more students from historically marginalized backgrounds. The program also provides opportunities for students from dominant social groups to learn about structural inequities to help address and dismantle systems that continue to marginalize underrepresented groups in research. A growing body of work shows the importance of connecting student identity and social motivations for STEM engagement within program designs in order to disrupt the typical structures of science education that reproduce social inequality (e.g., Carlone and Johnson, 2007; Garibay, 2015; Newman, 2011). Program designs that provide students with opportunities to explore and address their democratic and community values may contribute to the recruitment and retention of people who have historically been excluded from the biomedical field. Garibay (2018) argues that, along with gender, socioeconomic status (SES), racial/ethnic identification predict the personal values that may influence career choice:

With regards to racial/ethnic identification, STEM bachelor’s degree recipients who were Black or African American, Latina/o, and Asian American or Pacific Islander had significantly higher levels of social agency and values toward conducting meaningful research than their white counterparts, which connects to research showing an association between race/ethnic identification and democratic motivations for pursuing STEM majors.

(p. 368)

Garibay goes on to point out that if these students perceive a misalignment between their values and the values in STEM, this may discourage students from pursuing STEM majors (Garibay, 2018). The preliminary evaluation data reported here, especially the alumni data collected during the COVID-19 pandemic, highlight the importance of considering these personal and contextual dimensions of research experiences. Designing undergraduate research experiences that take into account the lived experiences of students from marginalized backgrounds can support students to meet their professional STEM goals and their commitments to their home communities and for social justice. This approach may reinforce their interest in joining and staying in the biomedicine and health sciences.

These results contribute to a continuing shift in program design of the Pathways Undergraduates program to create an environment that supports students to create more just and sustainable communities, and ideally will lead to increased retention of people from groups that have historically been excluded from the biomedical field. Here we identify four important design features of culturally responsive undergraduate research internships, that guide our work, based on these findings and related previous studies. These features may guide programmatic elements such as support for mentors, professional development planning for interns, and activities for cohort cohesion:

1. **Provide authentic research experiences:** Authentic research experience can help student interns both build science credentials that are important for career navigation in existing systems and position students “as producers of knowledge and culture who are capable of solving the problems facing our communities and our world” (Morales-Doyle, 2017). Students apply to the Pathways Undergraduates program to gain handson experience in biomedical research because of a desire to enter into a career in health/medicine and research suggests that these experiences are important for developing and sustaining interest (Estrada, 2014). Interns gained confidence and research skills by being treated as trusted contributors.
2. **Support community building**: A key focus of the Pathways Undergraduates program is connecting interns at a personal level with Fred Hutch scientists and staff through formal (mentoring) and informal interactions. Quality mentorship at Fred Hutch is an especially important component that can positively impact student outcomes, especially when mentors provide support for student goal attainment, sense of competence and identity, and building respect and connectedness (e.g., Estrada et al., 2018, 2021; Termini et al., 2021). Likewise, social activities are promoted to develop a support network among the interns and students in adjacent programs. Interns and alum appreciated opportunities to address the social and emotional aspects of succeeding in biomedical science, especially those they identified as having shared cultural backgrounds and values (Achinstein and Aguirre, 2008).
3. **Support social and emotional wellbeing in professional settings**: Pathways Undergraduates offers expanded sessions on developing resiliency and confronting imposter phenomenon (Clance and Imes, 1978), cognitive distortions, and stereotype threat (Murphy et al., 2020). Additional considerations to address systems in professional settings that have historically marginalized and/or excluded students from underrepresented groups may also be supportive for interns. For example, supporting bilingualism, offering flexible schedules, and helping interns connect their work to their home communities are just some ways that professional settings might reflect student values.
4. **Support students to situate themselves and their work in larger sociopolitical contexts, as well as in their home communities.** The alumni feedback reported here demonstrates how students are already making connections and situating how their experience fits into larger sociopolitical contexts–in part because of their internship experience. These findings make clear an opportunity for internship programs to build on what students are already doing to further support them as they recognize their own agency and strengthen their commitments to their home and professional communities.

### Applications for High School Programs.

The Pathways Undergraduates Program is unique in that it focuses on underrepresented first- and second-year college students with little research experience outside the classroom. With this regard, we feel that our findings and recommendations are also applicable to similar programs for high school students. Indeed, many of the programmatic approaches were developed in our longer running Summer High School Internship Program (SHIP) at Fred Hutch. For example, we adapt the following activities for high school interns:

#### Preparing Interns for the Next Steps of Their Academic Careers.

While Pathways Undergraduates focuses on post-college careers, SHIP focuses heavily on demystifying college selection, admissions, and success. Both programs bring rely on Fred Hutch staff and outside experts to address these concerns that are relevant to the career stage of the interns. Our programs are run concurrently, and the high school interns greatly benefit from near-peer interactions with undergraduates across the summer.

#### Providing Opportunities for High School Interns to Be Near-peer Mentors.

The Pathways Undergraduates gain leadership experience as near-peer mentors to the SHIP interns. Likewise, SHIP interns are given opportunities to be near-peer mentors to younger students during the summer. Pathways Explorers, also funded by the NCI R25 grant, is a two-week program at Fred Hutch for ninth- and tenth-grade students. SHIP interns meet with the Pathways Explorers several times to share their experience in the research lab. SHIP interns also serve as leaders in a half-day visit from the Seattle-based Rainier Scholars’ middle school program. In both cases, the high school students’ science identity is validated by their younger peers.

## CONCLUSION

The Pathways Undergraduates program model shows positive impacts for students in career awareness, science identity, and sense of belonging. During the pandemic, alumni responses indicated that the undergraduate research experience also supported their motivation to address health disparities and their sense of belonging in biomedicine by helping them to become credible resources in their home communities. The interns went from not only identifying as scientists, but also to becoming representatives of the biomedical research enterprise. These preliminary evaluation results offer the possibility that explicitly incorporating commitment to community and a justice-centered focus into research internships could enhance a sense of belonging in science, ultimately contributing to retaining more students in biomedicine. This possibility opens up new avenues of future research, both in future iterations of the Pathways Undergraduates program and in STEM internships generally. Future research that examines how undergraduate research experiences can better support students to make connections between STEM and personal or societal issues and concerns may ultimately help more students to see STEM careers as a way to support their social values and democratic commitments.

## Figures and Tables

**Table 1.** Pathways Undergraduates Program Components.

PROGRAM COMPONENTS	SUMMER (all interns)	ACADEMIC YEAR (optional)
**1. SCIENTIFIC**		
**Research Experiences**	Orientation/training week prepares students for mentored research 8-week immersion in Fred Hutch faculty research group with intern partner	Interns/alum may continue research with Fred Hutch faculty as circumstances permit.
**Research Seminars**	Fred Hutch faculty engage interns in seminars on Cancer Biology, Clinical Research, and Health Disparities Graduate students and scientific staff lead casual lunch-time sessions on research topics, methods, and day-to-day life as a scientist	
**2. PROFESSIONAL**		
**Mentorship**	Workshops on development of successful mentor relationships and building mentorship networks Mentoring with lab PI, researchers, and program staff Interns meet with graduate students and postdocs during casual “lunch and learn” sessions Undergrad interns serve as near-peer mentors to high school interns	Interns/alum continue contact with research mentors and program staff
**Career Development**	Panel sessions on Careers in Biomedical Research, Health/Medicine, Health Disparities and the Biotech Industry Workshops and practice on delivering a successful research presentation Workshops on the job hiring process, developing a CV, and writing a personal statement Intern-initiated informational interviews with Fred Hutch faculty	Writing support from Fred Hutch HR and program staff Academic and career guidance from program staff Career and research guidance from faculty
**Academic & Career Support**	Program staff supports interns as needed to meet their individual goals during the summer	Program staff supports interns/alum in meeting their individual goals during the academic year
**3. SOCIAL/EMOTIONAL**		
**Identity Development**	Identity-related workshops (Becoming a Resilient Scientist, Imposter Phenomenon, Overcoming Stereotypes, and Being a Minority at a Research Institution)	Research mentors and program staff advise interns/alum as needed
**Community Building**	Social events with undergraduate cohort and interns from high school summer program Lunch sessions with Fred Hutch community	Winter reunion and social gathering Intern-driven connections via social media

**Table 2.** 2018 & 2019 Pathways Undergraduate Intern Demographics.

Demographic	2018 cohort	2019 cohort	2018 & 2019 combined*
All Applicants	139	197	336

All Interns	22	33	49

Female	15 (68%)	18 (55%)	30 (61%)
Male	7 (32%)	15 (45%)	19 (39%)

NIH Underrepresented	95%	91%	92%

**URM Race/Ethnicity**	**18 (82%)**	**25 (76%)**	**38 (78%)**
Asian	4	3	7
Black/African American	8	14	20
American Indian/Alaska Native	2	3	3
White	1	3	4
Two or more	3	3	6
N/R or PNA	4	7	9
Hispanic/Latinx	6	9	13

**Low SES**	**11 (50%)**	**17 (52%)**	**24 (49%)**

**First Generation to College**	**12 (55%)**	**17 (52%)**	**25 (51%)**

**Disabled**	**1**	**5**	**5**

**Veteran**	**0**	**1**	**1**

Returning Hutch Intern (includes high school program alum)	11 (50%)	17 (52%)	26 (53%)

* Combined 2018 & 2019 numbers represent individual interns. Students who participated in both years are only counted once.

**Table 3.** Evaluation Data Collection Sources.

Focus	Data Sources	Evaluation Participants
1. Biomedical careers awareness and interest	Aug 2018 - Focus group with 8 new interns (in-person) Aug 2018 - Focus group with 8 returning interns (in-person)	2018 interns - 16 participants
	
2. Sense of belonging and identification as scientists	Aug 2019 - Focus group with 6 new interns (in-person) Aug 2019 - Focus group with 6 returning interns (in-person)	2019 interns - 12 participants

	March 2020 - Retrospective pre/post survey (online)	2018 & 2019 interns - 26 respondents

3. Effects of COVID pandemic on career interests and sense of belonging	March 2021 - Focus group (online)	2018 & 2019 interns - 6 participants

	Dec 2021 - Follow-up Survey (online)	2018 & 2019 interns - 6 respondents

**Table 4.** Participant Survey, McNemar Analysis.

Item 1: Awareness				Item 2: Knowledge			

Present (High rating: 3)	a = 0	b = 0	n1= 0	Present (High rating: 3)	a = 0	b = 0	n1= 0
Not present (Low ratings: 1, 2)	c = 22	d = 4		Not present (Low ratings: 1, 2)	n2 = 18	d = 8	
	n2 = 22		n = 26		n2 = 18		n = 26
McNemar’s (χ^2^ df=1)	22			McNemar’s (χ^2^ df=1)	18		
	p < 0.05				p < 0.05		
Item 3: Career Pathways				Item 4: Interest			

Present (High rating: 3)	a = 0	b = 0	n1= 0	Present (High rating: 3)	a = 10	b = 1	n1= 11
Not present (Low ratings: 1, 2)	c = 17	d = 9		Not present (Low ratings: 1, 2)	c = 10	d = 5	
	n2 = 17		n = 26		n2 = 20		n = 26
McNemar’s (χ^2^ df=1)	17			McNemar’s (χ^2^ df=1)	7.363		
	p < 0.05				p < 0.05		

p<0.05 for all items. McNemar’s test assumes there are two dichotomous tests (presence/non-presence of high score) and that each participant took both tests (pre and post). a, b, c, d compose a 2×2 contingency table of presence/non-presence in the pre and post tests.

## ABBREVIATIONS

Fred Hutch: Fred Hutchinson Cancer Center
HR: Human Resources
IACUC: Institutional Animal Care and Use Committee
MCB: Molecular and Cellular Biology
NCI: National Cancer Institute
NIH: National Institutes of Health
SES: Socioeconomic Status
SHIP: Summer High School Internship Program
SURP: Summer Undergraduate Research Program
The Hutch: Fred Hutchinson Cancer Center
URE: Undergraduate Research Experience
URM: Underrepresented Minority
YES: Youth Enjoy Science
